# Characteristics and Management of Autoimmune Disease-Associated Cerebral Venous Sinus Thrombosis

**DOI:** 10.3389/fimmu.2021.671101

**Published:** 2021-07-22

**Authors:** Baizhuo Zhang, Yue Lang, Weiguanliu Zhang, Li Cui, Fang Deng

**Affiliations:** Department of Neurology, Neuroscience Center, The First Hospital of Jilin University, Jilin University, Changchun, China

**Keywords:** autoimmune diseases, CVST = cerebral venous and sinus thrombosis, immunosuppressants, anticoagulants, treatment strategies

## Abstract

Cerebral venous sinus thrombosis (CVST) is a central nervous system disease characterised by thrombosis in cerebral venous or dural sinuses. Autoimmune diseases, a series of diseases caused by immune responses to autoantigens, are important causes of CVST. The most common diseases that lead to CVST are Behçet’s syndrome, systemic lupus erythematosus, antiphospholipid syndrome, and Sjögren’s syndrome. Each of these diseases have different clinical and imaging manifestations and treatment for CVST varies by aetiology. This review summarises the characteristics and the current management strategies for autoimmune disease-associated CVST and emphasises controversial therapeutic strategies to provide informative reference information for diagnosis and treatment. Risk factors of autoimmune antigens should not be neglected when unconventional CVST occurs, and both drugs and interventional therapy need further standardisation and discussion with more prospective clinical studies.

## Introduction

Cerebral venous sinus thrombosis (CVST) is a cerebrovascular disease presenting with headache, changes in intracranial pressure, and disturbance of consciousness caused by hypercoagulability and vessel wall damage. In this review, we aim to summarise the pathological mechanisms, clinical manifestations, therapeutic strategies, and prognosis of CVST caused by each disease. Further studies should be conducted regarding standard therapy and outcomes of autoimmune disease-associated CVST.

## Characteristics of Autoimmune Disease-Associated CVST

### Behçet’s Syndrome (BS)-Associated CVST

BS is a recurrent autoimmune disease characterised by systemic vasculitis. The main clinical manifestations are recrudescent oral ulcers and multi-system complications, including genital ulcers, ocular or skin lesions, nervous and haematological system syndromes, and arthritis (1). Complications of BS include central nervous system (CNS) and major macrovascular symptoms (2). There are two main types of CNS involvement: intra-axial neuro-BS (NBS), characterised by focal or multifocal parenchymal CNS lesions caused by venular inflammation (3), and extra-axial NBS, characterised by non-parenchymal CNS lesions mainly caused by intracranial vascular impairment (4), such as intravascular thrombosis, intracranial aneurysm, and extracranial aneurysm/dissection (5). Neurological symptoms are present in 3-33% of cases, and generally occur 2.5-6.5 years after BS diagnosis. CVST is one of the most important complications which accounts for 10-30% of extra-axial NBS (6). BS-related CVST was first reported in 1959 (7). The occurrence of CVST is strongly associated with systemic main vessel thrombosis. In addition, 64% of BS-related CVST patients also suffer from main vessel lesions (2). BS is a considerable cause of CVST, especially in certain Middle East and Mediterranean countries (3). The VENOST study, a multi-centre retrospective and prospective study of CVST, showed that 108 of 1144 CVST cases (9.4%) were caused by BS (8).

BS-related CVST is mostly identified 24 months after CNS complications appear. It is mainly characterised by intracranial hypertension and a progressive headache (8), followed by papillary oedema, cranial nerve VI palsy, and focal neurologic deficits or paresis/pyramidal tracts suggesting cerebral haemorrhagic infarction (9). The onset of CVST in most BS cases is subacute or chronic (4), which could help distinguish BS-related CVST from CVST of other aetiologies (3). Magnetic resonance venography (MRV) indicates that occlusions appear mostly in the superior sagittal and transverse sinuses, while the sigmoid and straight sinuses are less frequently involved. Isolated intracranial hypertension syndrome has been reported in certain cases without radiographic evidence. Moreover, no abnormalities are noted in cerebrospinal fluid (CSF) (10).

Endothelial cell impairment/activation was believed to be the only cause of thrombosis (3). However, recent studies (11, 12) showed that inflammatory responses could generate prothrombotic disorders, such as fibrinolysis (13) and platelet function alternation (14), which promote thrombosis and endothelial dysfunction (9). Furthermore, increasing neutrophil recruitment and activation in the acute phase of BS leads to the over-activation of apoptosis. Therefore, leukocyte oxidative stress and reactive oxygen species (ROS) generation derived by neutrophils contributes to fibrinogen post-translational modification and reduced fibrin susceptibility to plasminolysis in BS patients (12). Genetic factors, such as Factor V Leiden (*FVL*) gene mutation and methyltetrahydrofolate reductase (*MTHFR*) gene polymorphisms, may be related to BS thrombosis but are still controversial (15). Furthermore, hyperhomocysteinaemia has been confirmed as an independent risk factor of BS-related CVST (1), and increased homocysteine was found in 1 out of 5 patients (16).

BS-related CVST tends to have a favourable prognosis but delayed treatment will lead to neurological deficits (9). The prognosis of CVST with cerebral arterial infarction is relatively good while coexisting cranial nerve impairment or disturbance of consciousness refers to poor outcomes (modified Rankin score ≥2) (8). Some patients developed sequelae after proper treatment, such as optic nerve atrophy, blindness, or impaired vision (9).

### Systemic Lupus Erythematosus (SLE)-Associated CVST

SLE is a diffuse connective tissue disease which causes neuropsychiatric symptoms, referred to as neuropsychiatric lupus. CVST is one of the complications accompanying SLE. SLE with CVST was first reported in 1975 (17). Thrombotic events occur in 10-20% of patients with SLE (18), including thrombosis of the deep venous system of the extremities and the vena cava, while CVST is relatively infrequent. In a retrospective study from northern China (19), only 17 (0.36%) of 4747 patients with SLE suffered CVST. CVST caused by SLE does not show obvious differences compared to other aetiologies, and manifests with aggravated headache, visual field defect or diplopia, consciousness disorder, eyelid and conjunctival chemosis, and epilepsy (19). Lumbar puncture is conventionally performed, and intracranial pressure usually rises to more than 180 mmH_2_O or even 330 mmH_2_O. Almost half of all patients show higher protein content and one-third show an elevation of myelin basic protein in the CSF. MRV is the most important imaging examination for diagnosis and the transverse sinus is the most common place for thrombosis, followed by the superior sagittal and sigmoid sinuses. At least one thrombus was found in most patients surveyed (19).

SLE, a result of lupus anticoagulant (LAC) deposition and immune-mediated vasculitis, is also the main cause of SLE-related CVST. LAC and antiphospholipid (APL) antibodies can be detected in 40% of CNS-affected SLE patients (20), which prohibit the function of protein C (PC) and protein S (PS) and enhance thrombosis when interacting with vascular endothelial cells. Furthermore, fibrinolytic defects caused by hypercoagulability, functional alternation of antithrombin III, hyperfibrinaemia, and changes in coagulation are also pathogenic factors. *FVL* mutation can also be detected in some SLE patients (21). Cluster analysis was applied to identify SLE patients with similar autoantibody patterns and verify that the cluster of patients with anti-dsDNA/LAC/anticardiolipin (aCL) antibody was more closely related to arterial and/or venous thrombotic events and thrombocytopaenia, whereas it cannot test the causal association between cerebrovascular accidents and SLE (22, 23). Therefore, thrombophilia work-up, including assessments of PC, PS, LAC, antithrombin III, and *FVL* mutation, should be performed in patients with SLE and CVST. Moreover, Nishida et al. described an SLE patient with secondary aseptic meningitis who developed CVST after lumbar puncture. The causes were speculated as follows: 1) CSF reduction caused by lumbar puncture resulted in venous dilatation and high venous blood viscosity, 2) cerebral ptosis caused by CSF reduction led to venous and sinus occlusion, or 3) the onset of aseptic meningitis and steroid therapy directly caused the CVST (24). During follow-up with surviving patients, partial or total recovery and no recurrence of CVST or permanent neurologic deficits were observed (19).

### Antiphospholipid Syndrome (APS)-Associated CVST

APS is characterised by recurrent arterial and venous thrombosis, spontaneous abortion, circulating APL antibodies, and thrombocytopaenia, with positive laboratory examinations for both LAC and APL. APS is commonly secondary to SLE but can also be idiopathic, and CVST is less common than transient ischaemic aneurysm or stroke (25). APS with CVST as the initial presentation was first reported in 2009 (26). The main symptoms of APS-related CVST are headache (85%), visual defect (40%), and cognitive impairment (25%). The mechanism is still not fully understood, but it has been speculated that APL promotes the pre-thrombotic state by activating endothelial cells, platelets, and monocytes. In addition, anti-β2GP-1 antibodies and complement activation play an important role in thromboembolism (27).

### Sjögren’s Syndrome (SS)-Associated CVST

SS manifests as lymphocyte infiltration in salivary and lacrimal glands, therefore leading to simultaneous xerostomia and xerophthalmia. CNS complications among SS patients include aseptic meningitis, myelitis, microcirculation vasculitis related to white matter lesions, and encephalopathy (28). The proportion of SS patients suffering from neurological symptoms varies from 8% to 70% (29). Several retrospective studies have shown that 38% of SS patients are positive for APL, with LAC as the most common antibody. Among APL (+) patients, the incidence of stroke, deep venous thrombosis, and activated partial thromboplastin time elevation significantly increased, and thrombotic events were more frequent in LAC (+) patients than in LAC (-) patients. However, no CVST occurrence has been reported. In fact, CVST seldom happens in SS and it is well-understood that CVST can happen after the onset of SS. However, it is speculated to occur at the advanced stage (30). Clinical symptoms are almost identical to primary CVST, but 2-5% patients show no xerostomia or xerophthalmia (31). The most common site of thrombosis in SS-related CVST is the transverse sinus, but thrombi can also appear in the straight sinus, sagittal sinus, and Galen vein, among others (32). The detailed mechanism is not yet clear and a possible reason may be immune-mediated nerve impairment (32). Generally, the prognosis of CVST in SS is favourable, and there have been no clinical or radioactive recurrences in patients followed for 3 months to 5 years (33).

## Management of Autoimmune Disease-Associated CVST

### Anticoagulant Therapy

#### Conventional Anticoagulation

Intravenous heparin or low-molecular weight heparin followed with oral vitamin K antagonists (VKA) for 3-12 months according to various risk factors is recommended in the current guidelines of different countries (34–36). However, this treatment is controversial under some circumstances when autoimmune diseases are combined. There is not enough evidence for the safety and effectiveness of anticoagulant therapy in BS, although it has been applied to patients for a period of more than 3 years in western countries (37, 38). Thrombosis in BS is extensively suggested as a vasculitis-derived immune inflammatory response, and so anti-inflammatory therapy is regarded as sufficient. Moreover, aneurysm or pseudoaneurysm is usually detected during BS-related CVST and anticoagulant therapy could increase the risk of aneurysm rupture haemorrhage. However, anticoagulant therapy has been successfully applied in most reported cases with no malignant events. Nevertheless, imaging examinations, especially computed tomography pulmonary angiography, is strongly recommended in order to rule out possible aneurysms before anticoagulant therapy is applied (12). In APS-associated CVST, oral warfarin is recommended for an indefinite period in the reported cases since CVST patients with APS tend to experience thrombus recurrence, while there are still few researches to support the opinion (19).

#### Direct Oral Anticoagulants (DOACs)

There is a lack of high-level evidence of the safety and efficacy of DOAC treatment for autoimmune disease-associated CVST, and it has never been recommended in current guidelines as a major oral therapy. Nevertheless, several retrospective studies and case reports have used DOACs during APS treatment and revealed a higher risk of thrombosis recurrence than VKA. In addition, a recent meta-analysis of randomised controlled trials on DOACs in APS showed a higher risk for only arterial thrombosis recurrence rather than venous thrombosis, suggesting the therapeutic potential of DOACs for APS-associated CVST.

#### Anticoagulant Strategies When Combined With Thrombocytopaenia

Notably, thrombocytopaenia is a crucial complication in both SLE and APS, posing a dilemma for APS-associated CVST management. In fact, patients with APS-associated thrombocytopaenia have a higher risk of thrombosis than those without thrombocytopaenia, and therefore anticoagulant therapy seems more necessary when balancing the risks of thrombosis and haemorrhage. There is still a lack of guidelines towards CVST with APS-associated thrombocytopaenia, and it is generally recommended that treatment strategies conform to the idiopathic thrombocytopaenic purpura (ITP) guidelines since there are common characteristics between ITP and APS-associated thrombocytopaenia (39). Normal anticoagulation should be given when platelets ≥50×10^9^/L and the haemoglobin level is stable, while the dose is suggested to be halved when platelets <50×10^9^/L; glucocorticoids (GCs) or intravenous immunoglobulin (IVIg) should be used to elevate platelets to a safe level (30-50 ×10^9^/L) (40, 41).

### Glucocorticoid and Immunosuppressive Therapy

GCs and immunosuppressive therapy are well-known anti-inflammatory treatments. Azathioprine (AZP) is the top immunosuppressants for BS, while methotrexate, cyclophosphamide, and mycophenolate mofetil (MMF) are also recommended (42). Certain studies have emphasised the importance of immunosuppressants since anticoagulants alone do not attenuate the risk of venous thrombosis recurrence. Roriz et al. found no significant difference between immunosuppressants alone and the combination of anticoagulants and immunosuppressants (43). In addition, GCs and immunosuppressive therapy are applied in catastrophic APS, which is characterised by microvascular thrombosis leading to multiorgan failure (44), rather than isolated moderate-to-large vessel thrombosis in APS. The most commonly used strategy is the triple therapy of anticoagulants, GC and IVIg, and/or plasma exchange (45). When combined with diffuse alveolar haemorrhage, severe thrombocytopaenia, and haemolytic anaemia, immunosuppressants such as cyclophosphamide or rituximab are recommended (46). Hydroxychloroquine may be the first choice for SS (33).

### Endovascular Therapy (ET)

ET has been conventionally used in refractory CVST patients when large and extensive thrombi are unlikely to dissolve after anticoagulant treatment and has shown a reasonable safety profile (35). In a single-centre study of management of CVST patients with APS, endovascular mechanical thrombectomy, local endovascular thrombolysis, and internal jugular vein stenting were performed in patients who suffered from anticoagulant inefficacy, clinical deterioration, or severe neurological dysfunction (respectively), with satisfying outcomes (47). However, there are no reports of ET for the other autoimmune diseases mentioned, suggesting the need for further and broader studies.

## Conclusion

Autoimmune diseases are important risk factors for CVST, of which pathological mechanisms can be explained based on the mechanism of thrombosis, such as hypercoagulability or inflammation caused by vascular endothelial injury, while the specific pathologies of autoimmune disease-associated CVST have yet to be studied. It is easy to diagnose CVST according to clinical symptoms, case history, and radiography. When clinical manifestations like intracranial hypertension cannot be explained by conventional aetiologies of CVST, serum autoimmune antibodies and thrombohaemophilia tests should be considered. There are no standard or official guidelines for autoimmune disease-associated CVST treatment. Immune and anticoagulant therapy should be carefully analysed before use since there are advantages and disadvantages according to various complications and severities. Generally, the prognosis of autoimmune disease-related CVST is favourable with prompt treatment.

## Author Contributions

BZ and YL conceived the review, collected the data, wrote the paper, and contributed equally to this work. WZ helped analyse the data and revise the manuscript. LC and FD supervised the review and approved the final version of the manuscript. All authors contributed to the article and approved the submitted version.

## Funding

This work was supported by a grant from the National Natural Science Foundation of China (82071293) and the National Natural Science Foundation of China (82071351).

## Conflict of Interest

The authors declare that the research was conducted in the absence of any commercial or financial relationships that could be construed as a potential conflict of interest.
